# A cell polarity protein aPKCλ is required for eye lens formation and growth

**DOI:** 10.1016/j.ydbio.2009.10.010

**Published:** 2009-12-15

**Authors:** Yuki Sugiyama, Kazunori Akimoto, Michael L. Robinson, Shigeo Ohno, Roy A. Quinlan

**Affiliations:** a School of Biological and Biomedical Sciences, University of Durham, Durham, UK; b Department of Molecular Biology, Yokohama City University Graduate School of Medical Science, Yokohama, Japan; c Department of Zoology, Miami University, Oxford, OH, USA; d Advanced Medical Research Centre, Yokohama City University, Yokohama, Japan

**Keywords:** Lens, aPKC, Polarity, Cell junction, Proliferation

## Abstract

The organisation of individual cells into a functional three-dimensional tissue is still a major question in developmental biology. Modulation of epithelial cell shape is a critical driving force in forming tissues. This is well illustrated in the eye lens where epithelial cells elongate extensively during their differentiation into fibre cells. It is at the lens equator that epithelial cells elongate along their apical–basal axis. During this process the elongating epithelial cells and their earliest fibre cell derivatives remain anchored at their apical tips, forming a discrete region or modiolus, which we term the lens fulcrum. How this is achieved has received scant attention and is little understood. Here, we show that conditional depletion of aPKCλ, a central effector of the PAR polarity complex, disrupts the apical junctions in elongating epithelial cells so that the lens fulcrum fails to form. This results in disorganised fibre cell alignment that then causes cataract. Interestingly, aPKCλ depletion also promotes epithelial–mesenchymal transition of the lens epithelial cells, reducing their proliferation, leading ultimately to a small lens and microphthalmia. These observations indicate that aPKCλ, a regulator of polarity and apical junctions, is required for development of a lens that is the correct size and shape.

## Introduction

**Fig. S1:**
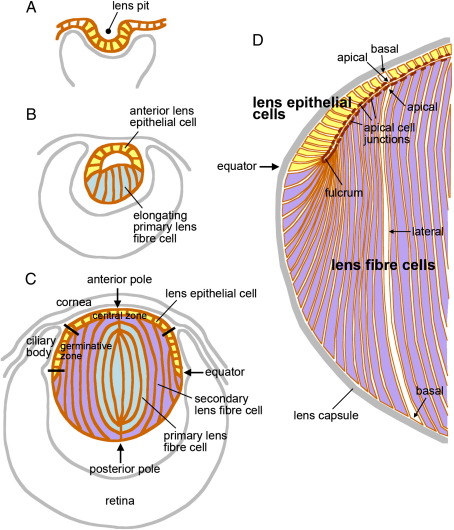
Lens development and structure. During embryogenesis, the lens arises from the surface ectoderm adjacent to forebrain, which thickens to form the lens placode and then invaginates to form the lens pit (A, E10.5). The lens pit finally separates from the ectoderm to form the lens vesicle (B, E11.5). Posterior epithelial cells of the lens vesicle start to elongate towards the anterior surface and finally fill the lumen of the lens vesicle (E12.5). These elongating cells are called primary lens fibre cells and constitute the central region of mature lens. Anterior epithelial cells of lens vesicle continue to proliferate as the lens expands whilst those near to the lens equator stop dividing and begin their program of differentiation. At the equator, these cells start to elongate and differentiate into secondary fibre cells (C). Newly formed fibre cells are added around the central mass of primary lens fibre cells. The secondary lens fibre cells are also polarised, with the basal membranes attached to the posterior lens capsule and the apical region contacting the apical membranes of the anterior lens epithelial cells (D). At their apical ends, neighbouring lens fibre cells are inter-connected by apical cell junctions, which are continuous to lens epithelial cell junctions.

The eye lens is an epithelial tissue derived from surface ectoderm and is composed of two types of cells, namely, epithelial and fibre cells (Fig. S1). The lens epithelial cells are located on the anterior surface of the lens as a monolayer and the lens fibre cells comprise the rest of the lens. The lens epithelial cells serve as progenitors for lens fibre cells and differentiate into lens fibre cells at the lens equator (Lovicu and McAvoy, 2005). Fibre cells have an extremely elongated cell shape and align along the anterior–posterior lens axis. Their ordered, anterior–posterior alignment is required for lens transparency. Any disruption of this organisation not only impairs light transmission but also affects lens function as well as causing cataract (Kuszak et al., 1994). Thus, to understand how a functional lens is made, it is important to understand the mechanism(s) that underpins lens formation, which includes the tissue organisation of the fibre cells.

Each lens fibre cell retains the same apical–basal polarity as the epithelial cells from which they are differentiated (Lo et al., 2000). At their anterior ends, the apical plasma membranes of the lens fibre cells appose the corresponding apical surfaces of lens epithelial cells. At the lens equator, the elongating epithelial cells and their earliest fibre cell derivatives appear to remain anchored at their apical tips, forming a discrete region or modiolus (Zampighi et al., 2000). This marks the start of the epithelial–fibre cell interface. The posterior basal ends of the fibre cells contact the inner surface of the posterior lens capsule and the interaction here with the extracellular matrix is mediated by integrins (Walker and Menko, 2008). At their apical ends, neighbouring lens fibre cells are inter-connected by adherens junctions (AJs) (Lo et al., 2000) and this close contact between cells is maintained along their lateral membranes by cadherin cell–cell junctions (Hatta and Takeichi, 1986; Lo et al., 2000) and at the apices of these membrane contacts by unique ball and socket interdigitations (Dickson and Crock, 1972; Hollenberg et al., 1976). The formation during differentiation of highly ordered, precisely aligned, lens fibre cells is likely to be dependent on the maintenance of their apical–basal cell polarity and/or their apical cell junctions. Recent knockout experiments show the importance of the AJ proteins to fibre cell organisation and lens formation (Cain et al., 2008; Cooper et al., 2008; Pontoriero et al., 2009). However, the mechanism(s) that orchestrates the alignment of these fibre cells and how this impacts lens size are not fully understood.

The formation of apical cell junctions and the establishment of apical–basal cell polarity in other epithelial cells is regulated by a protein complex comprising the atypical protein kinase C (aPKC) and the partitioning defective (Par) polarity proteins (Suzuki and Ohno, 2006). aPKC is a Ser/Thr kinase that forms a complex with Par3 and Par6, PDZ domain containing proteins. This protein complex localises to tight junctions (TJs) in epithelial cells. Inhibition of the activity of these proteins by overexpression of dominant negative constructs or by RNAi, delays formation of apical cell junctions comprising both AJs and TJs and allows diffusion of apical and/or basolateral membrane proteins into the lateral and/or apical domains, respectively (Suzuki and Ohno, 2006). Previously, we showed that bovine lens epithelial cells also express aPKC, Par3 and Par6 at apical cell junctions in intact lenses (Sugiyama et al., 2008), suggesting that the regulation of cell polarity and the formation of apical cell junctions in lens epithelial cells also occurs by aPKC/Par polarity proteins.

Here, we assess the involvement of the aPKC–Par system in lens formation and the alignment of differentiating lens fibre cells. We have generated lens-specific aPKCλ conditional knockout mice using a modified αA-crystallin promoter to target Cre just to the lens (Zhao et al., 2004). We show that aPKCλ depletion disrupted lens fibre cell organisation and induced cataractogenesis. We also found that aPKC knockout affected the cell cycle of lens epithelial cells. Our findings indicate that aPKC also has an important role in regulating lens growth.

## Materials and methods

### Mice

Mice harbouring a floxed *aPKC*λ gene in which exon 5 was flanked by loxP sequences (aPKCλ^*floxed*^) or mice with exon 5 of *aPKC*λ deleted (aPKCλ^*deleted*^) were generated by homologous recombination (Akimoto et al., unpublished data; Koike et al., 2005). MLR10-Cre mice, which use a modified αA-crystallin promoter designed to express Cre recombinase in all lens cells from the lens vesicle stage (E10.5) onward, have been described previously (Zhao et al., 2004). We analysed aPKCλ conditionally depleted lens phenotypes (conditional knockout; cko) by using aPKCλ*^floxed/floxed^*Cre^*+*^ or aPKCλ*^floxed/deleted^*Cre^*+*^ mice, both of which showed similar abnormalities with little animal to animal variation. Control mice had genotypes consisting of aPKCλ*^wt/floxed^*Cre^*+*^, aPKCλ*^floxed/floxed^*Cre^*−*^, or aPKCλ*^floxed/deleted^*Cre*^−^*, and displayed a normal phenotype identical to that seen in aPKCλ wild-type mice.

### Immunostaining

Dissected eyes or embryonic heads were fixed with 4% (w/v) paraformaldehyde/phosphate-buffered saline for paraffin wax-embedded sectioning (4–5 μm). Paraffin sections were hydrolysed and autoclaved at 120 °C for 10 min in 10 mM sodium citrate (pH 6.0) for an antigen retrieval step. Immunostaining was performed according to standard protocols using 10% (v/v) normal calf serum in TBST [10 mM Tris–HCl (pH 8.0), 150 mM NaCl, 0.05% (v/v) Tween 20] as a blocking reagent, and primary and secondary antibodies were diluted in 0.1% (w/v) bovine serum albumin/1.5% (v/v) normal goat serum/TBST, whereas goat serum was omitted from anti-goat antibody reaction. Antibodies used for immunohistochemistry in this study were as follows: rabbit polyclonal antibodies against CP49 (2981; Sandilands et al., 1995); rabbit antibodies against aPKC (C20, Santa Cruz sc-216), β-catenin (H-102, Santa Cruz sc-7199), ZO-1 (Zymed 61-7300), γ-tubulin (Sigma T3559), Par3 (Upstate 07-330) and active caspase-3 (Promega G7481); mouse antibodies against aPKCλ (TDL 610175), ZO-1 (Zymed 33-9100), E-cadherin (TDL 610181), N-cadherin (TDL 610920), BrdU (clone B44, BD 347580) and αSMA (clone 1A4, DAKO M0851 or Zymed 08-0160); and goat antibody against Kip2/p57 (Santa Cruz sc-1039). Images were captured using a BX50 fluorescent microscope (Olympus) equipped with a Sensys CCD camera (Photometrics), an LSM510 confocal microscope system (Carl Zeiss) or CSU10 spinning disc confocal microscopy (Yokogawa) attached to a Leica DMIRBE microscope. Some paraffin sections were stained with hematoxylin and eosin to visualize cellular morphology.

### BrdU labelling analysis

Pregnant mice were injected intra-peritoneally with BrdU (100 μg/g body weight) 1 h before euthanasia and embryo collection. Embryos were processed for immunostaining as described above and BrdU-positive cells were detected by mouse anti-BrdU antibody staining. For counting purposes, the lens epithelial layer was partitioned into two regions, central and germinative zones. We defined the central lens epithelial cells as being the cells that are situated in the proximity of the inner layer of the cornea. The germinative zone of the lens epithelial cells lies adjacent to the ciliary body and iris primordium (McAvoy, 1978).

## Results

### Loss of aPKCλ in lens cells induces small lens and cataract formation

**Fig. 1:**
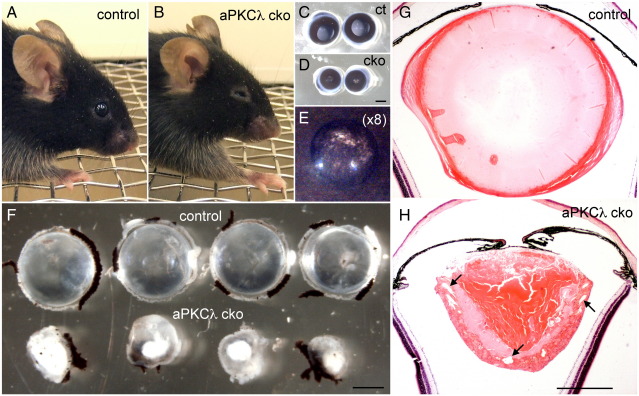
Eye lens-specific aPKCλ knockout causes microphthalmia (small eyes) and cataract formation. The eyes in a 2-month-old aPKCλ cko mouse (B) have a sunken, almond-like shape compared with the protruding, round eyes in a similarly aged control animal (A). Once removed, the eyeballs of aPKCλ cko (D) were smaller than controls (C) and had small opaque pupils with brown/black patches (E, an eight times enlarged view of D). In contrast to the large transparent control lenses (F, upper four lenses), aPKCλ cko lenses (F, lower four lenses) were small and had opaque lens nuclei, i.e., nuclear cataract. The HE stain section of an aPKCλ cko lens from a 4-month-old mouse shows disorganised fibre cell layers with abnormal spaces (H, arrows). This contrasts the more homogenous fibre mass of the section of a similarly aged, control lens (G). Scale bars: 1 mm for (C), (D), and (F) and 500 μm for (G) and (H).

Since homozygous knockout of aPKCλ causes early embryonic lethality before lens induction (Akimoto et al., unpublished data), we used conditional depletion to remove aPKCλ expression from the lens. The MLR10 transgenic mice express Cre recombinase from E10.5 onwards in lens cells (Zhao et al., 2004). In contrast to control mice (Fig. 1A), aPKCλ conditional knockout (cko) mice have small, almond-shaped eyes (Fig. 1B). The eyeballs of the cko mice were smaller than control mice and had small opaque pupils speckled with black material (Figs. 1C–E). The lenses of cko mice were substantially smaller than those of control mice and developed nuclear cataracts (Fig. 1F). The fibre cell mass was well organised as seen in the cross-section of control lenses (Fig. 1G), but in contrast the cko lenses showed severely disorganised fibre cell layers (Fig. 1H). Pigmented cells that appeared to be continuous with the iris covered the anterior surface of the cko lenses (Fig. 1H). These pigmented cells are likely to be the basis for the black-speckled appearance of the pupils (Fig. 1E). The abnormal eye appearance and associating smaller lens are already evident when mice open their eyes around postnatal day 14 (data not shown). These data indicate that the conditional depletion of aPKCλ in the lens has disrupted lens development resulting in a small lens and finally a cataract. Therefore aPKCλ is essential both for a functional lens and for normal lens growth.

### aPKCλ localisation in lens cells

**Fig. S2:**
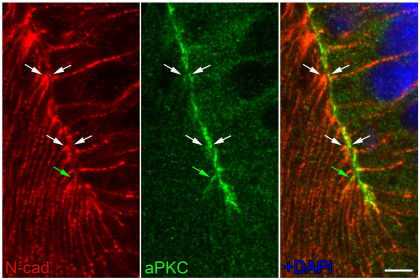
Loss of aPKC from apical cell junction during fibre differentiation (E16.5). Optical sections (same field in Fig. 2P) around the equator region of a control lens showing the apical cell junctions of the epithelial (DAPI stained nuclei; blue) and elongating secondary fibre cells. N-cadherin (red) shows apical cell junctions as well as lateral membranes of both epithelial and fibre cells, whereas the apical accumulation of aPKC (green) is detected only in the epithelial cells. Apical membranes of epithelial and fibre cells are distinct especially at the regions indicated (white arrows). The last aPKC signal found in fibre cells is indicated (green arrow). Scale bar shows 5 μm.

**Fig. 2:**
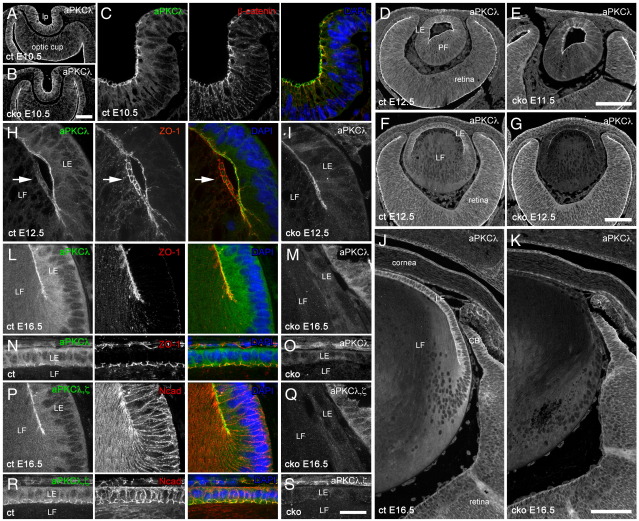
Depletion of aPKCλ protein in conditional knockout lenses. Confocal fluorescence microscopy images of lens sections immunostained as indicated. In aPKCλ cko lenses, a reduction in aPKCλ was not detected at lens pit stage (E10.5; cf. A and C with B) although signal intensity began to show a decrease at E11.5 (cf. D and E), but partial loss from both lens epithelial and fibre cells was evident at E12.5 (G) compared to the control (F), when primary fibre cell elongation was complete and secondary fibre cells had begun to differentiate. All the detectable aPKCλ was lost from lens cells by E16.5 (cf. J and K; L and M). aPKCλ was localised at the apical cell junctions of lens pit cells (C; an enlarged image of lens epithelial cells at the lens pit stage shown in A) and in central epithelial cells at later stages (H, L, and N). aPKCλ, however, was not found at the apical ends of the lens fibre cells that were about to complete elongation (H, white arrows), suggesting aPKCλ is lost from the apical cell junction upon completion of primary fibre cell elongation. The immunostaining of aPKCλ in control lenses also indicates the presence of cytoplasmic aPKCλ in both lens epithelial and fibre cells (see both F and G and J and K). An antibody that detects both aPKCλ and ζ isoforms showed similar patterns to those obtained with an aPKCλ specific antibody (P–S). ct, control; cko, conditional knockout; lp, lens pit; LE, lens epithelial cell; PF, primary fibre cell; LF, lens fibre cell, CB, ciliary body. Scale bars: 50 μm in (A) and (B); 20 μm in (C), (H), (I), and (L) to (S); 100 μm in (D) to (G), (J), and (K), respectively.

To determine the mechanism(s) involved in these lens defects, we examined the cellular distribution of aPKCλ protein during lens development (Fig. 2). During embryogenesis, the lens arises from the surface head ectoderm, which thickens to form the lens placode and then invaginates to form the lens pit (Lovicu and McAvoy, 2005; see Fig. S1). During lens development, aPKCλ is normally detected at the apical junctions of the epithelial cells in the lens pit at E10.5. These are regions where ZO-1, the tight junction marker (data not shown), and β-catenin, the adherens junction marker, also accumulate (Figs. 2A and C). aPKCλ remains at the apical cell junctions of the anterior lens epithelial cells during primary lens fibre cell elongation, which occurs around E12 (Fig. 2D), after closure of the lens vesicle at E12.5 (Figs. 2F and H). The accumulation of aPKCλ at the apical junctions of the elongating primary lens fibre cells was clearly present at this stage (Fig. 2D), but it was lost by E12.5 from these junctions upon closure of the lens vesicle (Fig. 2H). Note that the signal for aPKC was barely detectable at the apical tips of last remaining fibre cells that have still to make contact with the overlying lens epithelial cells (Fig. 2H, arrows). In contrast, ZO-1 was clearly located at the apical cell–cell junctions at the tips of these fibre cells as seen by the typical cobblestone-like pattern usually seen for the lens epithelium. At E16.5, the signals for aPKCλ and ZO-1 were still colocalised in the lens epithelial cells (Figs. 2J, L, and N). Since aPKCλ was detected at the apical cell junctions of lens epithelial and elongating primary fibre cells, but was significantly reduced in the elongated primary fibre cells (Fig. 2H) and was apparently absent from secondary fibre cells (Fig. 2N; see Fig. S2), we suggest that aPKCλ is gradually lost from the apical cell junctions of lens fibre cells during their differentiation.

In aPKCλ cko lenses, aPKCλ staining appeared normal at the lens pit stage (Fig. 2B), but was weakened by E11.5 when primary fibre cell elongation occurs (Fig. 2E). At E12.5, aPKCλ staining was clearly reduced from both apical cell junctions and the cytoplasm of lens epithelial and fibre cells (Fig. 2G). At these stages the signal intensity of the surrounding retina and cornea was unchanged and this served as a useful positive control (Fig. 2G). At higher magnification (Fig. 2I), the cytoplasmic signal for aPKCλ at E12.5 was almost absent, whilst the signal intensity was maintained along the epithelial–fibre cell interface, that is, at the apical cell junctions of aPKCλ cko lenses. By E16.5, even this staining of aPKCλ was undetectable (Figs. 2K, M, and O).

In addition to the aPKCλ specific antibody, we also used anti-aPKC antibodies to detect aPKCζ, a close isotype of aPKCλ, as well as aPKCλ (Figs. 2P–S). The staining patterns for both antibodies were indistinguishable at the apical cell junctions of lens epithelial cells where both ZO-1 and N-cadherin (another AJ marker) also localise. In aPKCλ cko lenses, the signal for aPKCλ/ζ was also lost (Figs. 2Q and S), suggesting that aPKCζ was not expressed in the lens after E16.5.

### Disorganised fibre cells and thinner lens epithelial layer in embryonic aPKCλ depleted lenses

**Fig. 3:**
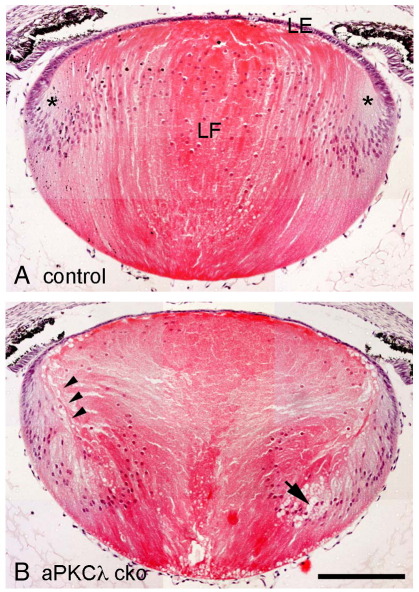
Embryonic defects observed in aPKCλ cko lenses (E18.5). HE staining of lens sections. The shape of the aPKCλ cko lens appeared normal (B), but the lens fibre layer was distorted and the apical tips of elongating fibre cells (arrowheads) were detached from the anterior lens epithelial cells. Abnormal extracellular spaces can be seen (large arrow) in the lens fibre cell mass. The anterior lens epithelial cell layer was thinner in aPKCλ cko lens than in the control lens. Asterisks in the control lens (A) indicate the lens equator where the adherent apical tips of the differentiating lens fibre cells form the lens fulcrum, a region where the tips of the differentiating epithelial cells and their earliest fibre cell derivatives accumulate. Scale bar shows 200 μm.

Next we examined the lens phenotype at E18.5, a stage when aPKCλ can no longer be detected by immunofluorescence microscopy in the lenses of aPKCλ cko animals. The control lens shows a monolayer of anterior lens epithelial cells and well-aligned lens fibre cells (Fig. 3A). While the aPKCλ cko lenses were in equivalent size and maintained the gross lens morphology, comprising an anterior lens epithelial layer and a fibre cell mass, the epithelial layer was noticeably thinner and fibre cell alignment was severely disrupted (Fig. 3B). Interestingly, the most cortical (i.e., youngest) lens fibre cells appear to have lost their apical connections at the modiolus (Zampighi et al., 2000). This is the point where lens epithelial cells begin to elongate rapidly and adopt fibre cell characteristics. We refer to this region from now on as the lens fulcrum (Fig. S1D) to indicate the turning point for lens fibre cells in their differentiation process as they become organised to contribute to the lens mass. Compared to the lens fulcrum in a control where the apical ends of epithelial cells and their fibre cell derivatives colocalise (Fig. 3A, asterisks), the apical tips of the fibre cells in the aPKCλ cko lens appear detached from the anterior lens epithelial cells (Fig. 3B, arrowheads). The inner fibre cells of the aPKCλ cko lens were clearly distorted and occasionally, abnormal extracellular spaces were detected (Fig. 3B, arrow). The nuclei of the cortical fibre cells of aPKCλ depleted lens were distributed more basally without forming the so-called “bow” arrangement at the equator as observed in control lenses. These observations indicate that aPKCλ depletion affects both lens epithelial and lens fibre cells during embryonic lens development.

**Fig. 4:**
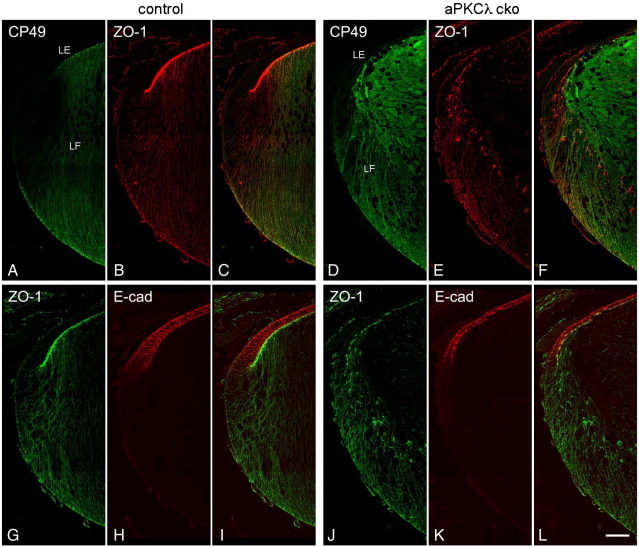
Lens fibre cell differentiation is not affected in aPKCλ cko lenses. In control lens (A–C), CP49 was expressed in fibre cells, but not in lens epithelial cells. CP49 expression was maintained in aPKCλ cko lenses (D–F), although the fibre cell alignment was disrupted and the signal at the plasma membranes is not as strong as in controls. E-cadherin is normally expressed in the lens epithelial cells and is lost at the equator when the cells initiate fibre differentiation (G–I). A similar differentiation-associated decrease of E-cadherin expression was detected in aPKCλ cko lenses (J–L) as is observed in control lenses. LE, lens epithelial cell; LF, lens fibre cell. Scale bar shows 50 μm.

We then asked if the fibre cell disorganisation of aPKCλ cko lens was related to the suppression of fibre cell differentiation. CP49 (BFSP2) is an intermediate filament protein induced in lens fibre cells during differentiation (Ireland and Maisel, 1984; Sandilands et al., 1995). We observed that aPKCλ cko lenses maintained the expression of CP49 in elongating fibre cells as observed in the control lenses (Figs. 4A–F), albeit the plasma membrane localisation appeared to be less distinct in the cko lens sections. Control lens epithelial cells express E-cadherin, but its expression was suppressed during fibre cell differentiation (Figs. 4G–I; Nose and Takeichi, 1986). This pattern and change in E-cadherin was retained in aPKCλ cko lenses (Figs. 4J–L). These results indicate that at least some protein expression patterns typical of fibre cell differentiation were maintained in aPKCλ cko lenses.

### Loss of aPKCλ in lens impairs formation of the lens fulcrum and leads to disorganised fibre cell alignment

To elucidate the primary cause of the lens phenotype in aPKCλ conditional knockouts, we examined the phenotype of earlier embryonic stages. No obvious defects were seen in aPKCλ cko lenses at E10.5, a stage when aPKCλ protein expression appeared normal (Fig. 2B). When aPKCλ depletion was becoming obvious at E11.5, primary fibre cell elongation had already commenced and the anterior cavity of the lens vesicle was closed normally in the aPKCλ cko (Figs. 2E and G).

**Fig. 5:**
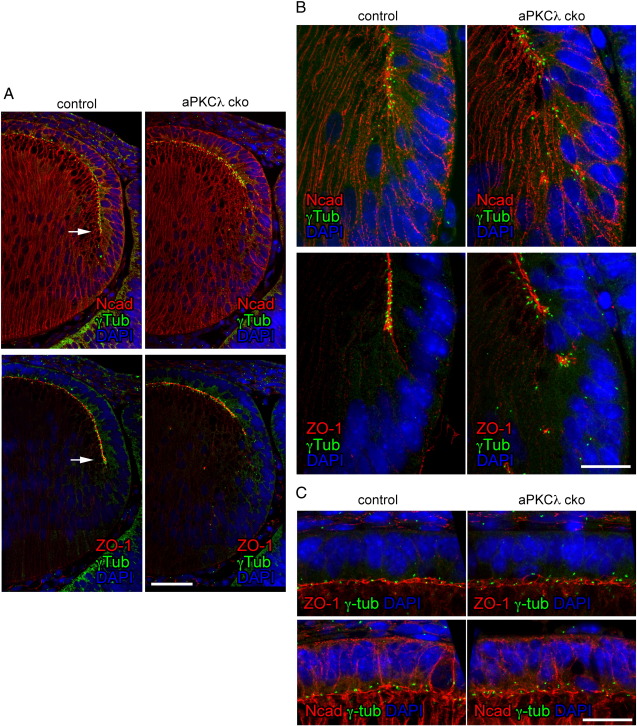
The lens fulcrum is no longer present at the lens equator in aPKCλ cko lens (E12.5 and E13.5). In a control lens, differentiating lens epithelial cells move their apical–basal axis through approximately 90° at the lens equator. This is achieved by the apical tips of the lens cells forming the equatorial lens fulcrum (arrows in A). It marks the start of the epithelial–fibre cell interface. At low magnification image (A, E12.5), a shortened epithelial–fibre cell interface is seen in an aPKCλ cko lens. In an enlarged view of a control lens (B, E13.5), γ-tubulin dots (green) are visible along the interface both in epithelial and fibre cell sides. In aPKCλ cko lenses, the apical tips of elongating fibre cells (N-cadherin, ZO-1 and γ-tubulin labelling) were clearly dissociated from the ends of newly formed fibre cells at the epithelial–fibre interface. This indicates a defect in the formation of the lens fulcrum. (C, E13.5) Lens epithelial cells at the anterior pole maintained apparently normal apical cell junctions at this stage. Scale bars are 50 μm in (A) and 20 μm in (B) and (C), respectively.

The earliest phenotypic change in aPKCλ cko lenses was detected at E12.5 and was manifested by the misalignment of the apical tips of elongating secondary fibre cells (Fig. 5A). In control lenses, an interface is normally formed between the apical surfaces of the lens epithelial cells and the underlying fibre cells. At the lens equator, the apical tips of the lens cells remained focused at the lens fulcrum and stayed fixed even as these cells left the epithelial cell layer. This fulcrum can be identified by N-cadherin and ZO-1 staining (Fig. 5A, arrows) and is marked by γ-tubulin puncta, which also located close to the apposing apical membranes of both lens epithelial and fibre cells (Fig. 5A, controls). In aPKCλ cko lenses, the interface between epithelial and fibre cell layer was also present, but it was much shorter and the apical ends of the cells were not as well structured (Fig. 5A, cko). Both the N-cadherin staining of the lateral membranes and the large number of stray γ-tubulin foci in the fibre cells of the cko lens illustrate this point (Fig. 5A, cko). At higher magnification, it can be seen that the N-cadherin staining was lost from the epithelial–fibre interface and became more posterior (Fig. 5B, cko; images shown in B are of lenses from E13.5 as representative data). This dislocation indicates that the apical tips of elongating fibre cells do not maintain their association with epithelial cells at the equatorial fulcrum and the modiolus is abnormal. Newly differentiating lens fibre cells failed to align properly and were distorted, giving rise to a disorganised lens fibre mass that became worse as development progressed. In summary, aPKCλ is required for the successful completion of the lens fulcrum at the lens equator during fibre cell elongation. It appears that this is essential for correct fibre cell alignment.

Interestingly, the apical tips of detached lens fibre cells were also strongly positive for ZO-1, N-cadherin and γ-tubulin puncta (Fig. 5B, cko). This shows that the apical membranes of the aPKCλ cko lens fibre cells still retain some of their expected properties for this membrane compartment (see also Figs. 4E and J showing ZO-1 accumulations on the apical tips of dislocated fibre cells of aPKCλ cko lenses at E18.5). In addition, the basal membranes of these cells appeared very similar to those in control lenses with no obvious abnormalities. Whilst these observations suggest that lens fibre cells in aPKCλ cko lenses retain some aspects of their apical–basal polarity, this needs some qualification. The apical cell junctions in the central lens epithelial cells of aPKCλ cko lenses did not exhibit any obvious defect at E13.5 (Fig. 5C, at E13.5), suggesting that the apical cell junctions in equatorial epithelial cells might be more vulnerable to aPKCλ depletion than the central lens epithelial cells.

### aPKCλ depletion decreases proliferation rate of lens epithelial cells

Lenses from aPKCλ cko mice are abnormally small, i.e., microphthalmic (Fig. 1). One of the reasons for this could be a fibre cell elongation defect, but it could also be a lens epithelial cell proliferation defect. This is because lens epithelial cells in the germinative zone form the meridional epithelial cells that differentiate into lens fibre cells to sustain lens growth throughout life (Lovicu and McAvoy, 2005). Lens growth will therefore cease if the epithelial cells fail to replenish the fibre cell precursors in the meridional pool at the lens equator.

**Fig. 6:**
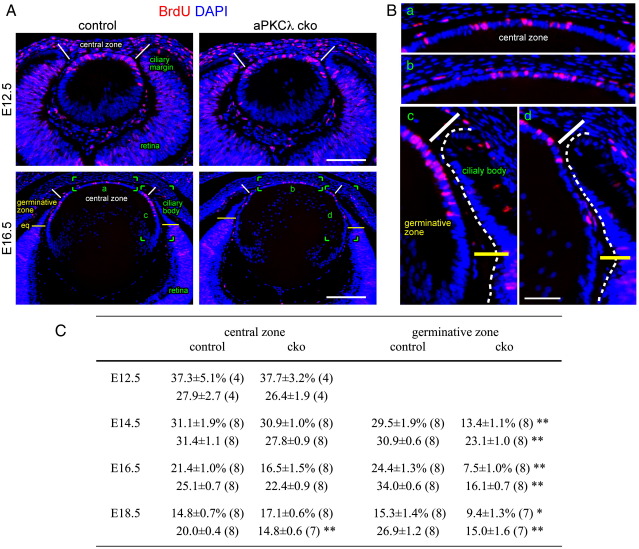
Decreased BrdU incorporation in the germinative zone of aPKCλ cko lenses. (A and B) S-phase nuclei were labelled with BrdU (red) for 1 h followed by DAPI staining (blue). The marked areas (a–d) of E16.5 lenses in (A) are enlarged in (B). Similar numbers of BrdU-positive cells were observed in the central zone of the lens epithelium in control and aPKCλ cko lenses. In the germinative zone, however, there were significantly less BrdU-positive cells in the aPKCλ cko lens than in an age-matched control. (C) BrdU incorporation rate (%, upper row) and cell number in a set length (100 μm, lower row). BrdU incorporation rate was decreased in the germinative zone of aPKCλ cko lenses. Both labelled and unlabelled cells were counted and the incorporation rate is presented as the percentage of cells that were labelled. Means ± SEM (*n* =   number of slides counted); ⁎*P* < 0.01 and ⁎⁎*P* < 0.0001 by two-tailed Student's *t* test. Cells counted: central zone control, 3031; cko, 2567; germinative zone control, 2071; cko, 1172. Epithelial layer length examined: central zone control, 12,309 μm; cko, 12,078 μm; germinative zone control, 6797 μm; cko, 6733 μm. Scale bars: 100 μm for E12.5 and 200 μm for E16.5 in (A) and 50 μm in (B).

To explore this possibility, the influence of aPKCλ on the cell cycle was analysed by BrdU incorporation. BrdU-positive lens cells in the central and germinative zones of the lens epithelium were counted (Figs. 6A and B). We found a sharp reduction in BrdU incorporation in the germinative zone of aPKCλ cko lenses at E14.5 compared to wild-type lenses, which was maintained in E16.5 and E18.5 cko lenses (Fig. 6C). Consistent with this reduction of BrdU incorporation, total cell number/unit length was significantly reduced in the germinative zone. By contrast, no statistically significant difference in BrdU incorporation was seen between control and aPKCλ cko lenses in the central epithelial cells during these stages of development (Fig. 6C). A significant reduction in cell number, however, was detected in this central zone at E18.5, and cell numbers tended to be lower than controls from E14.5 onwards. Thus this significant decrease in cell number in the central zone at E18.5 might reflect a compensatory response to the drastic reduction in cell number and BrdU labelling in the germinative zone of the epithelial cell layer. Collectively, these data suggest a reduction in proliferative activity is caused by aPKCλ depletion and this appears to occur specifically in the germinative zone.

The germinative zone was not well developed at the E12.5 stage so we only counted BrdU-positive cells of the central zone in the lens epithelium (Fig. 6A). At this stage we found no significant difference between control and cko lenses (Fig. 6C). We also measured BrdU incorporation rate for the whole lens cells including both epithelial cells and primary fibre cells at E12.5. There was no difference between control and aPKCλ cko lenses (control 12%, 110/935, *n* =   4; cko 12%, 122/1028, *n* = 4).

**Fig. S3:**
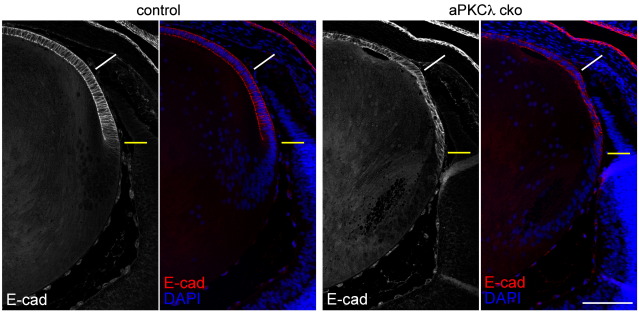
E-cadherin expression in aPKCλ cko lens (E16.5). An optical section produced by immunofluorescence confocal microscopy of control and aPKCλ cko lenses immunostained with an epithelial cell marker, E-cadherin (red) and DAPI (blue). White and yellow lines indicate the distal and proximal boundaries, respectively, of the ciliary body. In both control and aPKCλ cko lenses, the germinative zone epithelial cells adjacent to the ciliary body are positive for E-cadherin expression, which finishes at the equator. Scale bar shows 100 μm.

The differences we observed were not due to any relocation of the germinative zone or because cells in the germinative zone had started to differentiate. Both possibilities could be excluded because we detected E-cadherin-positive epithelial cells in the germinative zone and also saw suppression of E-cadherin expression at the equator in aPKCλ cko lenses, as also seen for controls (Fig. S3). We also did not detect any ectopic BrdU-positive fibre cells in E14.5 to E18.5 lenses from aPKCλ cko mice (data not shown). Taken together these observations indicate that aPKCλ cko lenses show a normal proliferation rate up to the completion of primary fibre cell elongation. There is then a decrease in proliferation in the germinative zone of the lens epithelium when secondary fibre cell formation commences. The data suggest that aPKCλ is required for normal proliferative activity in the germinative zone of the lens.

**Fig. 7:**
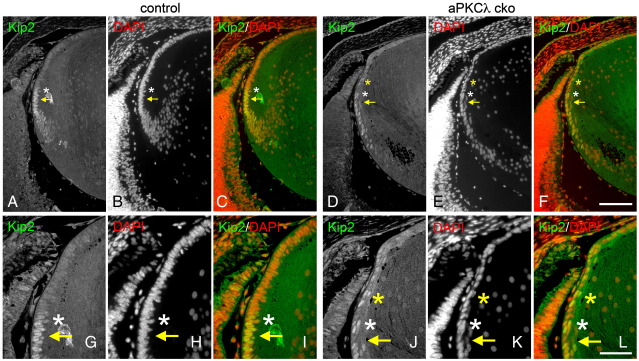
A postmitotic marker is ectopically induced in proliferation zone of aPKCλ cko lens (E16.5). p57/Kip2 is a cyclin-dependent kinase inhibitor expressed in postmitotic cells. In control lens (A–C, G–I), Kip2 was induced just anterior (white asterisks) to the equator (arrows) where the lens epithelial cells withdraw from their cell cycle and begin to differentiate into fibre cells. In aPKCλ cko lenses (D–F, J–L), Kip2 was detected more anterior to the equator (yellow asterisks). Scale bars: 100 μm in (A) to (F) and 50 μm in (G) to (L).

A reduction in cell proliferation might be accounted for cells withdrawing prematurely from the cell cycle in the germinative zone of aPKCλ cko lenses and we do find some evidence for this. The expression of the postmitotic marker, Kip2/p57, was similar at the equator of control (Figs. 7A–C and G–I, white asterisks; Zhang et al., 1997) and aPKCλ cko lenses (Figs. 7D–F and J–L, white asterisks). Ectopic Kip2 signal was, however, detected in some cells in the germinative zone of aPKCλ cko lenses giving a mosaic pattern (Figs. 7D–F and J–L, yellow asterisks). We also detected abnormal Kip2 induction in the germinative zone of aPKCλ cko lenses at E18.5 (data not shown).

**Fig. S4:**
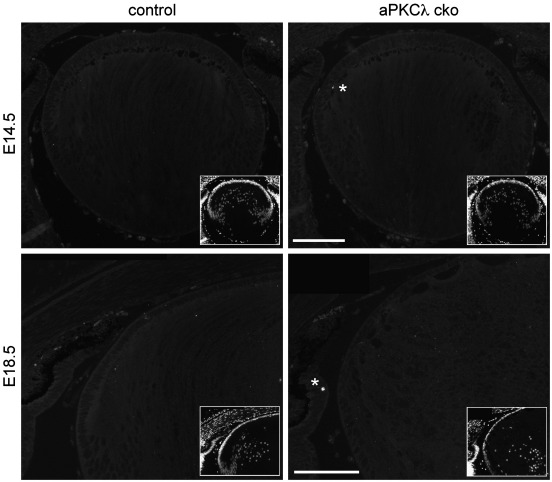
Apoptosis induction in aPKCλ cko lenses (E14.5 and E18.5). Optical sections produced by immunofluorescence confocal microscopy of control and E14.5 and E18.5 aPKCλ cko lenses immunostained with an antibody to active caspase 3. The insets show nuclei of each section counterstained with DAPI. Only a single cell was identified in aPKCλ cko lens sample at E14.5 with caspase 3 staining (asterisk), but no positive cells were found either in aPKCλ cko lens at E18.5 or E14.5 and E18.5 control mouse lenses. A positive caspase 3 signal can be seen in a cell in the ciliary body in aPKCλ cko image at E18.5 (asterisk). Since signal detection intensity was increased to visualise background level signals, some autoimmunofluorescence was seen in red blood cells in surrounding ocular tissues. Scale bars indicate 100 μm.

A reduction in cell proliferation could also be explained by increased apoptosis in the aPKCλ cko lenses. We found a very low frequency of apoptosis in aPKCλ cko lenses at E14.5 (Fig. S4) and E16.5 (data not shown), with the detection of only one apoptotic cell. At E18.5, no apoptotic cells were detected (Fig. S4). We suggest, therefore, that it is premature withdrawal from the cell cycle rather than increased apoptosis that causes reduced proliferation in the germinative zone of aPKCλ cko lenses.

### Induction of EMT in aPKCλ depleted lenses

Lens epithelial cells can transdifferentiate into myofibroblast-like cells, a process that is detected by the expression of α-smooth muscle actin (αSMA). Lens epithelial cells can undergo EMT as a consequence of cataract surgery or injury (Hales et al., 1994; Schmitt-Graff et al., 1990). This proceeds via the TGFβ/Smad pathway due to the fact that the aqueous humour contains the TGFβ ligand (Saika, 2006).

**Fig. 8:**
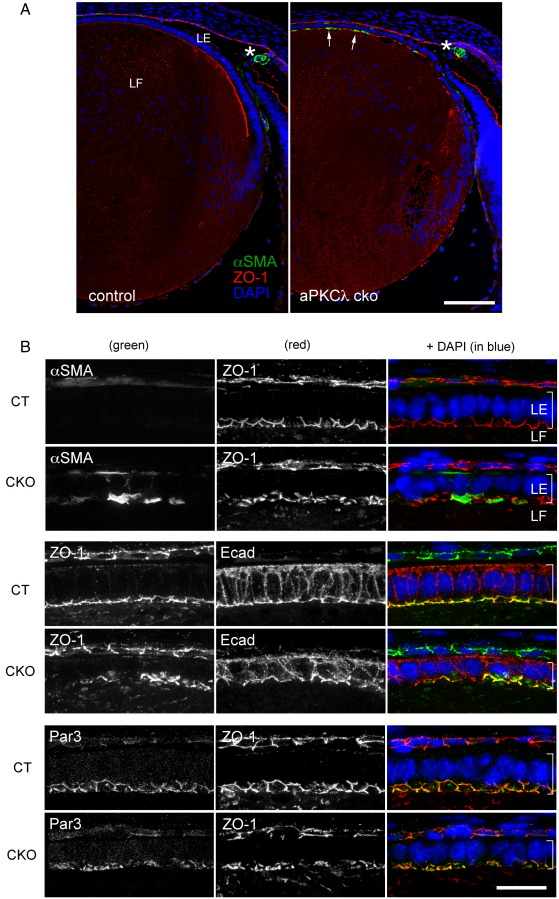
Loss of aPKCλ causes αSMA induction in lens epithelial (E16.5). (A) Alpha smooth muscle actin is not normally expressed in lens epithelial cells and therefore a marker for EMT. From E16.5 onwards, αSMA expression (green) was detected in epithelial cells of aPKCλ cko lenses (arrows), indicating EMT induction. Iris myocytes normally express αSMA and serve as a positive control (asterisk). (B) Enlarged view of lens epithelial cells around the lens anterior pole at E16.5. A z-stack of confocal images shows the cobblestone-like staining of apical cell junctions. The lens epithelial cells of aPKCλ cko mice are thinner and the apical cell junctions are fragmented or have disappeared. LE, lens epithelial cell; LF, lens fibre cell. Scale bars: 100 μm in (A) and 20 μm in (B).

We detected an increased expression of αSMA in lens epithelial cells of aPKCλ cko mice from E16.5 onward (Figs. 8A and B). By P14, nearly all the epithelial cells in aPKCλ cko lenses were αSMA positive (data not shown). Fibronectin, another EMT marker, was not detected in 1-month-old lenses, but was strongly positive in 4-month-old lenses of aPKCλ cko mice (data not shown). At this stage, lens epithelial cells were multilayered (data not shown). In turn, E-cadherin was suppressed in fibronectin-positive multilayered-lens epithelial cells (data not shown). These data are consistent with EMT being a prominent feature in aPKCλ cko lenses.

The disassembly of cell junctions and the loss of cell polarity are well-known hallmarks of EMT (Thiery and Sleeman, 2006). Though we did not detect obvious disruption of cell junctions in the central lens epithelial cells of aPKCλ cko lenses at E13.5 (Fig. 5C), we speculated they were impaired at the time of αSMA induction. As expected, apical cell junctions revealed by ZO-1 or E-cadherin were fragmented or had disappeared from cell contacts of lens epithelial cells at E16.5 when αSMA was detected (Fig. 8B). The polarised localisation of Par3, another cell polarity protein, a binding partner of aPKCλ, at cell junction was also affected (Fig. 8B). These data indicate that aPKCλ is required to suppress EMT in lens epithelial cells and perhaps the maintenance of apical cell junction and/or cell polarity.

## Discussion

To understand the mechanism behind the formation of a highly-organised anterior–posterior alignment of fibre cells in the lens, we focused on the role of aPKC, a regulator of apical–basal cell polarity and apical junction formation. We found that aPKCλ depletion in the lens impairs the formation of the lens fulcrum during fibre cell differentiation at the lens equator. We also found that aPKCλ is required for both lens epithelial cell proliferation and EMT inhibition.

### aPKCλ is required for formation of the lens fulcrum at the lens equator

Although it has been suggested that the modiolus at the equator is essential to the organisation and alignment of secondary lens fibre cells (Zampighi et al., 2000), we show for the first time that aPKCλ is central to this process and the formation of the lens fulcrum. In aPKCλ cko lenses, the apical cell junctions in the cells at the lens equator became dislocated and the apical tips of the elongating secondary fibre cells separated from their neighbours and from the apposed, overlying epithelial cells. This was particularly important at the lens fulcrum point where normal contact between the apical ends of the lens epithelial cells and the fibre cells was disrupted. Fibre cells failed to elongate normally, resulting in fibre cell disorganisation.

The disruption of apical cell junctions in aPKCλ cko lenses was consistent with the established function of aPKC in regulating apical cell junction formation in epithelial cells. Therefore, we suggest that aPKCλ depletion initially disrupted apical cell junctions at the lens equator and then caused the detachment of the apical tips of the elongating fibre cells from the lens fulcrum. It is, however, also possible that depletion of aPKCλ prevents fibre cell elongation and caused detachment of the apical tips of fibre cells, preventing the formation of the lens fulcrum. In this case, however, the basal ends of the fibre cells might also be expected to have been detached from lens capsule, but this was not evident in aPKCλ cko lenses. We also have excluded the possibility that aPKCλ cko disrupted the expression of other fibre-specific proteins because CP49, a fibre cell specific intermediate filament protein required for fibre cell organisation and lens function (Sandilands et al., 2003), was expressed normally. We thus prefer the explanation that aPKCλ determines the turning point in lens cell differentiation and is responsible for the formation of the lens fulcrum by regulating apical cell junction formation/maintenance at the lens equator.

The mode of cell junction disruption was somewhat unexpected. The initial defect was detected at the equator in elongating fibre cells at E12.5 whereas the cell junction defects in the central epithelial cells only became obvious at a much later stage (E16.5). One explanation for this is that the cell junctions were disrupted first between cells at the lens equator because of the greater mechanical stresses experienced here. Alternatively, the active rearrangement of the cell junctions at the lens fulcrum may require a rapid turnover of aPKC to accompany cell junction reorganisation as these cells leave the epithelial cell layer and differentiate into fibre cells.

Equally surprising was the fact that fibre cells in aPKCλ cko lenses seemed to retain some cell polarity. Both ZO-1 and N-cadherin were still concentrated at the apical tips of these cells and the γ-tubulin foci also maintained their apical surface location. Our data suggest this was not due to compensation by aPKCζ, as it was not detected in aPKCλ cko lens cells (Figs. 2Q and S). Rather, it may be a common defect of lumen-enclosing epithelial monolayers because a similar defect has been observed in three-dimensional MDCK epithelial cell cultures in vitro. When grown inside an extracellular matrix gel, MDCK cells form cysts, a spherical monolayer of polarised cells that enclose a central lumen (O'Brien et al., 2002). RNAi suppression of Pals1 results in aberrant cysts with multiple lumens (Straight et al., 2004). Whilst ZO-1 was diffusely stained in these cysts, the cortical actin cytoskeleton and E-cadherin patterns were unchanged as too was the distribution of and an apical membrane marker, GP135, which still localised to luminal surfaces. A similar result was obtained following cdc42 RNAi (Martin-Belmonte et al., 2008). Thus the defect observed in aPKCλ depleted lenses could have a similar cause. The apical tips of the fibre cells often clustered, perhaps in an attempt to form a mini lumen (Fig. 5B). Notice in this example γ-tubulin puncti formed a circle around ZO-1 staining in aPKCλ cko lens fibre cells. Though the lumen is buried and no longer exists in wild-type lenses, a similar mechanism to form and maintain a lumen may still be part of the fulcrum formation at the lens equator by the epithelial and fibre cells.

### aPKC is involved in maintenance of fibre cell progenitors

In aPKCλ depleted lenses, the cell proliferation rate was reduced in the germinative zone as shown by the reduction in BrdU labelling (Fig. 6C) and ectopic expression of a postmitotic marker, Kip2, was evident in this region (Fig. 7). Since no significant increase in apoptosis in the germinative zone was detected (Fig. S4), we suggest the decreased cell number in the germinative zone of aPKCλ cko lenses was partially due to inappropriate withdrawal from the cell cycle. Lens epithelial cells in the germinative zone are a reservoir for future lens fibre cells. Thus any reduction in epithelial cell number in the germinative zone will reduce both the fibre cell number and the lens size. Our data suggest aPKCλ is needed for lens growth by maintaining the epithelial cell number in the germinative zone of the epithelium.

Why the epithelial cells in the germinative zone and not in those epithelial cells in the central zone were affected is an intriguing question. aPKCλ was removed from both groups of epithelial cells and indeed, cell junctions were disrupted in all these cells by E16.5. In wild-type postnatal lenses, proliferation is restricted to the lens epithelial cells in the germinative zone and this suggests that some mitogens secreted from the ciliary body maintain their proliferation. In contrast in embryonic lenses, no difference in proliferation rate was observed in either the central or germinative zone epithelial cells (Fig. 6C). These data suggest that proliferation is regulated by different signalling pathways in the central and germinative zones of embryonic lenses to explain the aPKCλ cko effects in the germinative zone. aPKC can regulate cell cycle via several different signalling pathways (Akimoto et al., 1998, 1996; Diaz-Meco et al., 1993; Sanz et al., 1999), suggesting some of these pathways are active only in germinative zone epithelial cells. In zebrafish retina, loss of aPKC increases proportion of neuroepithelial progenitors that undergo symmetric proliferative cell divisions, but reduces asymmetric neurogenic cell divisions that produce a daughter cell to differentiation (Baye and Link, 2007). So far, existence of cells that undergo asymmetric cell divisions has not been revealed in lens epithelial cells, but such a population could potentially occur in the germinative zone and would potentially be affected by loss of aPKC.

### EMT induction as a phenotype in aPKCλ cko lens epithelial cells

Induction of EMT in lens epithelial cells has long been recognised since it is commonly detected following cataract surgery (Saika, 2006). Cataract surgery removes the fibre cell mass. Some lens epithelial cells are always left attached to the lens capsule and these frequently undergo EMT with the resulting myofibroblast-like cells proliferating and causing lens capsule wrinkling and/or secreting extracellular matrix deposits on the lens capsule. These changes obstruct light transmission and cause so-called secondary cataract, the major complication of cataract surgery.

EMT in the lens has been linked to a wound-healing reaction and can be also produced by experimental injury (Saika et al., 2004). TGFβ, which is present in aqueous humour that bathes the lens epithelial cells, stimulates the wound-healing response and activates the downstream Smad pathway to induce EMT (de Iongh et al., 2005). EMT can be also induced by the depletion of aPKCλ from a lens. Cell junction disassembly and the loss of cell polarity are hallmarks of early EMT (Thiery and Sleeman, 2006). Furthermore, the disruption of cell junctions is also a key step in TGFβ-induced EMT (Masszi et al., 2004). In aPKCλ cko lens epithelial cells, we observed the disruption of apical cell junctions and the mislocalisation of polarity proteins. Recently, it has been reported that conditional depletion of β-catenin and E-cadherin, as well as β1-integrin in lens epithelial cells, also induced EMT (Cain et al., 2008; Pontoriero et al., 2009; Simirskii et al., 2007). Basal membrane integrity has been also recognised as a critical factor for EMT induction (Thiery and Sleeman, 2006). Thus proteins that are essential for lens epithelial cell integrity, like aPKCλ, appear to be required to prevent EMT.
